# Tumor Protein 53-Induced Nuclear Protein 1 Enhances p53 Function and Represses Tumorigenesis

**DOI:** 10.3389/fgene.2013.00080

**Published:** 2013-05-13

**Authors:** Jeyran Shahbazi, Richard Lock, Tao Liu

**Affiliations:** ^1^Histone Modification Group, Children’s Cancer Institute Australia for Medical ResearchRandwick, Sydney, NSW, Australia; ^2^Faculty of Science, School of Biotechnology and Biomolecular Sciences, UNSW Science, University of New South WalesKensington, Sydney, NSW, Australia; ^3^School of Women’s and Children’s Health, UNSW Medicine, University of New South WalesRandwick, Sydney, NSW, Australia

**Keywords:** TP53INP1, p53, protein phosphorylation, apoptosis, autophagy

## Abstract

Tumor protein 53-induced nuclear protein 1 (TP53INP1) is a stress-induced p53-target gene whose expression is modulated by transcription factors such as p53, p73, and E2F1. TP53INP1 gene encodes two isoforms of TP53INP1 proteins, TP53INP1α and TP53INP1β, both of which appear to be key elements in p53 function. In association with homeodomain-interacting protein kinase-2 (HIPK2), TP53INP1 phosphorylates p53 protein at Serine-46. This enhances p53 protein stability and its transcriptional activity, leading to transcriptional activation of p53-target genes such as p21 and PIG3, cell growth arrest and apoptosis upon DNA damage stress. The anti-proliferative and pro-apoptotic activities of TP53INP1 indicate that TP53INP1 has an important role in cellular homeostasis and DNA damage response. Deficiency in TP53INP1 expression results in increased tumorigenesis, whereas TP53INP1 expression is repressed during early stages of cancer by factors such as miR-155. This review aims to summarize the roles of TP53INP1 in blocking tumor progression through p53-dependant and p53-independent pathways, as well as the elements which repress TP53INP1 expression, hence highlighting its potential as a therapeutic target in cancer treatment.

## Introduction

The TP53 gene encodes for the p53 protein which modulates target gene expression, regulates cell cycle progression and apoptosis, and functions as a tumor suppressor. p53 has been described as “the guardian of the genome,” due to its role in conserving stability by preventing the occurrence of mutation in the genome (Strachan and Read, 1999).

One of the key target genes of p53 is tumor protein 53-induced nuclear protein 1 (TP53INP1). TP53INP1 is expressed in many tissues upon exposure to various stress agents, and encodes two nuclear isoforms, TP53INP1α and TP53INP1β, both of which appear to be key elements in p53-mediated cell cycle arrest and apoptosis in different cell types (Tomasini, 2003). As a tumor suppressor, TP53INP1 has been reported to be down-regulated in cancers from different organs (Jiang et al., 2006; Gironella et al., 2007; Shibuya et al., 2010).

Tumor protein 53-induced nuclear protein 1 gene localizes to human chromosome 8q22 (Nowak et al., 2005), which shows sequence conserved with the A1–A2 of the murine chromosome 4 where the mouse TP53INP1 has been mapped (Carrier et al., 2000). Sequence analysis by the HUGO Gene Nomenclature Committee has revealed that stress-induced protein (SIP), p53-dependent damage-inducible nuclear protein 1 (p53DINP1), and thymus-expressed acidic protein (TEAP) are all in fact TP53INP1.

Tumor protein 53-induced nuclear protein 1 was first cloned and characterized in an attempt to identify pancreatic genes induced by the cellular stress acute pancreatitis in mouse, using a quantitative fluorescent cDNA microarray hybridization approach (Tomasini, 2001). The mouse TP53INP1 gene is almost 20 kb pairs in length with five exons. The exon 4 of 28 base pairs is alternatively spliced to generate two transcripts which translates into two nuclear proteins of 18 and 27 kDa, SIP^18^ and SIP^27^ (Tomasini, 2001) corresponding to TP53INP1α and TP53INP1β respectively (Tomasini, 2003). TP53INP1α and TP53INP1β proteins differ in their C-terminal region and can promote apoptotic cell death when overexpressed (Tomasini, 2001). Both TP53INP1α and TP53INP1β are rapidly and strongly induced in pancreatic acinar cells during the acute phase of pancreatitis and the exposure to various stress agents such as UV, DNA base damaging, ethanol, heat shock, and oxidative stress.

In this review, we aim to summarize the mechanisms through which TP53INP1 blocks tumor progression via p53-dependant and -independent pathways, and the mechanisms through which TP53INP1 gene expression is suppressed in cancer. Additionally, we will discuss the diverse functions of TP53INP1 in cancer such as induction of autophagy and repression of tumor cell migration, and highlight its potential as a therapeutic target in cancer treatment.

## TP53INP1 Functions as a Tumor Suppressor and Induces Apoptosis through Phosphorylating p53 at Serine-46

Multiple lines of evidence suggest that TP53INP1 gene expression is modulated by p53. Firstly it has been shown that cells with deleted, mutated, or inactive p53 are unable to activate TP53INP1 gene expression in response to stress agents (Tomasini et al., 2002). Secondly mouse embryo fibroblasts transformed with rasV12/E1A has shown stronger induction of TP53INP1 mRNA expression by activating p53-dependent pathway, compared to fibroblasts without p53 activity (Tomasini et al., 2002). Both observations suggest that TP53INP1 gene expression is activated by p53 in response to stress or transformation in cells expressing wild-type p53.

**Figure 1:**
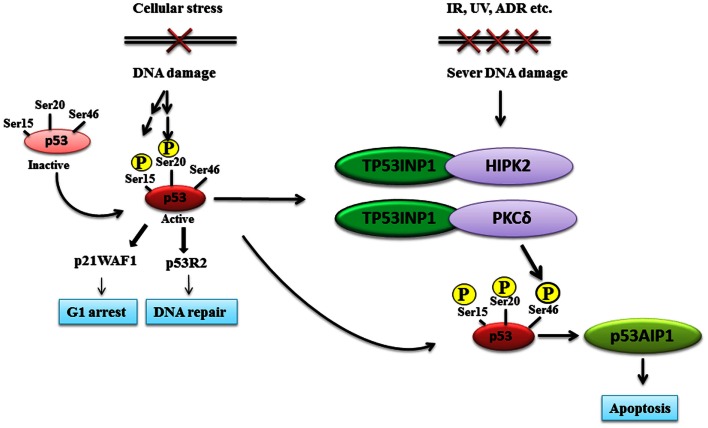
**TP53INP1 induces p53 protein phosphorylation at Ser-46 and apoptosis after DNA damage**. Upon initial DNA damage, p53 is phosphorylated at Ser-15 and Ser-20, stimulating the binding of p53 to the promoter regions of a subset of genes such as the G1 arrest gene p21, the DNA repair gene p53R2 and p53 negative regulators. If DNA damage is severe, TP53INP1 forms protein complexes with the Ser-46 kinase HIPK2 and PKCδ, leading to p53 protein phosphorylation at Ser-46, induction of p53AIP1 gene transcription and apoptosis.

Tumor protein 53-induced nuclear protein 1 phosphorylates p53 at Serine (Ser)-46 by forming protein complexes with the protein kinase homeodomain-interacting protein kinase-2 (HIPK2) or protein kinase C δ (PKCδ) (Figure 1). HIPK2, a member of a novel family of nuclear serine/threonine kinases, co-localizes with p53 and PML-3 in the nuclear bodies and is activated after irradiation with ultraviolet. HIPK2 directly interacts with p53 and phosphorylates p53 at Ser-46, leading to p53-target gene transcription and the activation of p53-dependent apoptosis pathway (D’Orazi et al., 2001; Hofmann et al., 2002). Further analysis of subcellular distributions showed that p53, HIPK2, TP53INP1α, and TP53INP1β all localize into the pro-myelocytic leukemia nuclear bodies, PML-NB, which are cell cycle-regulated nuclear structures appearing as punctate foci in interphase nuclei (Tomasini, 2003). Such co-localization facilitates the protein interactions by positioning the resulting complex near its site of action (Tomasini, 2003). Importantly, both TP53INP1α and TP53INP1β in association with HIPK2 regulate p53 transcriptional activity on p21, PIG3, and BAX promoters, induce G1 cell cycle arrest and increase p53-mediated apoptosis (Tomasini, 2003).

In another study, Yoshida et al. (2006) have demonstrated that PKCδ, another kinase from the family of nuclear serine/threonine kinases, also associates with p53 and mediates its phosphorylation at Ser-46 upon exposure to genotoxic agents, hence promoting p53-mediated apoptosis in cellular response to DNA damage (Yoshida et al., 2006). Moreover, PKCδ functions as a protein kinase and physically binds to TP53INP1 upon genotoxic stresses, leading to the formation of the PKCδ-TP53INP1 protein complex to regulate p53 protein phosphorylation at Ser-46 as well as p53-induced apoptosis (Yoshida et al., 2006).

The induction of growth inhibition and apoptosis is one of the most important tumor suppressive functions of p53. The challenge to find the exact mechanisms of p53-dependent apoptosis remains ongoing. In 2000, it was shown by Oda et al. (2000) that phosphorylation of p53 at Ser-46 by the p53-target gene; p53 regulated apoptosis inducing protein 1 (p53AIP1), could specifically regulate the induction of apoptosis. p53AIP1 was originally isolated as a p53-target gene using yeast enhancer trap system that allowed direct cloning of p53-binding sequence from human genomic DNA in order to isolate p53-target genes. p53AIP1 gene expression is strongly inducible by DNA damage in a p53-dependent manner, and is specifically induced after the phosphorylation of p53 protein at Ser-46, leading to apoptosis (Oda et al., 2000). Importantly, TP53INP1 induces p53 phosphorylation at Ser-46 and p53AIP1 expression, whereas the inhibition of TP53INP1 expression clearly impairs p53 phosphorylation at Ser-46 and p53AIP1 expression. Therefore, TP53INP1 is required for p53 phosphorylation at Ser-46, the induction of p53AIP1 and apoptosis (Okamura et al., 2001) (Figure 1).

As p53 is the most important tumor suppressor and p53 mediates cellular stress responses which are disrupted during tumorigenesis, it is important to further understand the mechanisms through which TP53INP1 interacts with HIPK2 and PKCδ kinases and phosphorylates p53 protein at Ser-46. As there are only handful studies demonstrating that TP53INP1 forms a complex with these two kinases to this date, it is important to understand what other co-factors are involved in the protein complexes and how these co-factors promote the formation of the protein complexes and the phosphorylation of p53 protein at Ser-46.

## TP53INP1 Induces Growth Inhibition and Apoptosis in a p53-Independent Manner through p73

It has been shown that in the absence of p53, TP53INP1 gene transcription can be strongly induced by p73, a p53 homolog. p73 is up-regulated in response to cisplatin, gamma-irradiation, and the oncogene E1A (Das et al., 2003; Hershko et al., 2005), activates p53-target gene transcription by binding to p53-responsive elements at p53-target gene promoters (Obad et al., 2004), and consequently induces cell cycle arrest and/or apoptosis (Jost et al., 1997; Kaghad et al., 1997). When p53-deficient mice are induced to suffer from acute pancreatitis or p53-deficient embryonic fibroblasts are treated with cisplatin, a strong DNA damaging agent, p73 activates TP53INP1 gene transcription, causes cell cycle arrest and apoptosis in a p53-independent manner (Tomasini et al., 2005). Using cells from p53-deficient mice, Tomasini et al. (2005) have demonstrated that the activation of the TP53INP1 gene promoter by p73 requires the presence of the p53-responsive element which is located between 1364 and 1239 base pairs upstream of TP53INP1 transcription start site. This suggests that p73 overexpression can directly activate TP53INP1 gene transcription through direct binding to the promoter region of TP53INP1.

Conversely, TP53INP1 alters the transactivation capacity of p73 on several p53-target genes, including TP53INP1 itself, demonstrating a functional association between p73 and TP53INP1 (Tomasini et al., 2005). Importantly, when overexpressed in p53-deficient cells, TP53INP1 activates p73, inhibits cell growth, and promotes cell death as assessed by cell cycle analysis and colony formation assays, hence the activation of TP53INP1 could potentially prevent tumor development (Tomasini et al., 2005). It is worth mentioning that TP53INP1 is able to stimulate p53 activity at much higher level compared to p73 activity.

## Induction of TP53INP1 Gene Expression by E2F1 can be either p53 Dependent or Independent

E2F1 is a transcription factor, which induces apoptosis via both p53 dependent and independent mechanisms. E2F1 controls the expression of a vast number of genes that are essential for progression from G1 to S phase (Hanahan and Weinberg, 2011). A study by Hershko et al. (2005) have shown that excessive activity of E2F1 results in increased expression of TP53INP1 as well as several other co-factors such as the apoptosis stimulating proteins of p53 (ASPP) family member ASPP1 and ASPP2, and the pro-apoptotic JMY. Although it is well documented that E2F1 can up-regulate p53 expression (Hershko et al., 2005; Polager and Ginsberg, 2009), ectopic expression of E2F1 in p53-null human H1299 lung adenocarcinoma cells results in an increase in TP53INP1 mRNA (Zemskova et al., 2010). This indicates that E2F1-mediated up-regulation of TP53INP1 can occur in a p53-independent manner. Moreover, chromatin immunoprecipitation assays with primers targeting −415 bp to −121 bp region of the TP53INP1 gene promoter, confirms that E2F1 directly binds to TP53INP1 gene core promoter and activates TP53INP1 gene transcription (Hershko et al., 2005). Given that E2F1 can play a major role in cell cycle progression and apoptosis, it could provide very important information to further investigate the underlying mechanisms responsible for E2F1-induced TP53INP1 expression.

## Modulation of TP53INP1 Expression by Inflammatory Mediators

Inflammation contributes to the tumor microenvironment by providing the tumor with essential factors for proliferation, survival, tumor cell migration, and invasion. Inflammatory mediators play important roles in tumor initiation and development in certain cancer types, such as prostate cancer (De Marzo et al., 2007). TP53INP1 expression has recently been found to be enhanced in prostate cancer cells after treatment with the pro-inflammatory mediators tumor necrosis factor α and interleukin 6, indicating that TP53INP1 overexpression could be involved in inflammation-mediated prostatic carcinogenesis (Giusiano et al., 2012). In this scenario, overexpression of TP53INP1 actually results in increased tumorigenesis, contradicting with the tumor suppressor characteristic of TP53INP1 as described in various other cancers, suggesting a tissue specific function. Perhaps, TP53INP1 can act either as a tumor suppressor gene or an oncogene depending on the tissue type or the tumor microenvironment.

## Micro RNAs Reduce TP53INP1 mRNA Expression

MicroRNAs (miRNAs) are a new class of small (21–23 nucleotides) non-coding RNAs. They function as post-transcriptional regulators of gene expression through base-pairing to complementary sites on their target mRNAs and are involved in carcinogenesis. Multiple lines evidences demonstrate that TP53INP1 expression can be regulated by miRNAs at the post-transcriptional level. For example, Yeung et al. (2008) have shown that the miRNAs miR-93 and miR-130b, which are up-regulated in HTLV-1–transformed human T-cell lines, target the 3′ un-translated region (3′UTR) of TP53INP1 mRNA, and that knocking-down the miRNAs significantly increases TP53INP1 mRNA expression and reduces proliferation and survival of the HTLV-1 infected/transformed cells (Yeung et al., 2008). In CD133(+) liver tumor-initiating cells, TP53INP1 is a direct target of miR-130b which promotes cell growth (Ma et al., 2010).

Tumor protein 53-induced nuclear protein 1 expression can also be modulated by miR-17-5p and miR-17. While miR-17-5p suppresses cell growth and promotes apoptosis of cervical cancer cells, TP53INP1 expression is reduced by miR-17-5p (Wei et al., 2012). In chronic lymphocytic leukemia (CLL) cells, up-regulation of miR-17∼92 family miRNAs by Toll-like-receptor-9 agonists is preceded by a transient induction of the proto-oncogene Myc, and forced expression of miR-17, a major member from the miR-17∼92 family, reduces TP53INP1 expression and protects cells against apoptosis (Bomben et al., 2012).

Additionally, TP53INP1 RNA can be targeted and down-regulated by miR-155 and miR-125b. miR-155 is overexpressed in pancreatic cancer cells and interacts with TP53INP1 mRNA at its 3′UTR (Gironella et al., 2007), whereas miR-125b is overexpressed in type II endometrial carcinoma cells and contributes to the malignancy of type II endometrial carcinoma, possibly through down-regulation of TP53INP1 expression (Jiang et al., 2011). Regulation of TP53INP1 expression by miR-125b can be potentially important for more effective therapy, since various studies have established miR-125b as an oncogene or tumor suppressor gene in difference types of human tumors (Bousquet et al., 2010; Zhang et al., 2011; Bhattacharjya et al., 2013).

## TP53INP1 Induces Autophagic Cell Death

Autophagy is an important physiological response that is strongly induced during cellular stress, mainly under nutrient deficiency. Autophagy is basically a recycling event whereby the cellular organelles will be engulfed within the autophagosome and broken down upon contact with lysosome, consequently generating metabolites used for biosynthesis and energy metabolism to support the survival of cancer cells in the nutrient limited environment (Hanahan and Weinberg, 2011). Autophagy-related protein 5 (ATG5), beclin-1 and the light chain of the microtubule-associated protein 1 (LC3, a member of ATG8 family proteins) are key players in autophagic cell death (White, 2012).

**Figure 2:**
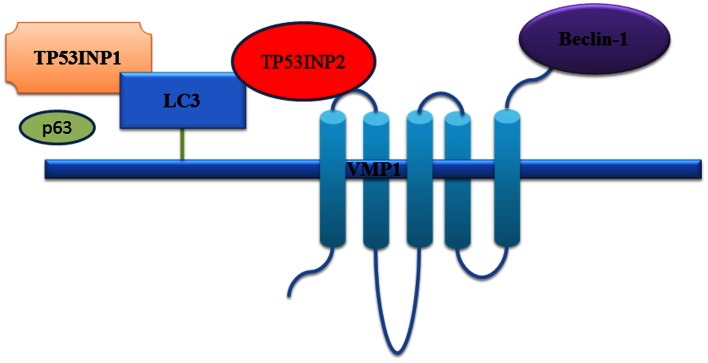
**TP53INP1 and TP53INP2 co-operatively induce autophagy**. Under autophagy-inducing stress such as starvation, TP53INP2 translocates from the nucleus to the autophagosomes, interacts with VMP1, and recruits the critical autophagy regulator LC3 and beclin-1 (Nowak and Iovanna, 2009). TP53INP1 interacts with LC3 via a functional LC3-interacting region with affinity higher than p62, and displaces p62 from autophagosomes.

While mainly a nuclear protein, TP53INP1 re-localizes into autophagosomes during autophagy, where TP53INP1 interacts with LC3 via a functional LC3-interacting region (Sancho et al., 2012; Seillier et al., 2012) (Figure 2). As TP53INP1 binds to LC3 with affinity higher than p62, the suppression of which promotes tumorigenesis (Mathew et al., 2009), TP53INP1 can partially displace p62 from autophagosomes and thus modify the composition of autophagosomes (Seillier et al., 2012).

Studies with mice with non-functional beclin-1 gene has shown increased susceptibility to cancer, due to the impairment in autophagy which requires to be circumvented during tumor development (Levine and Kroemer, 2008; White and DiPaola, 2009). Importantly, silencing beclin-1 or ATG5 or inhibiting caspase activity, which is necessary for the induction of autophagy, significantly decreases TP53INP1-induced cell death, indicating the effect of TP53INP1 in inducing autophagic cell death (Sancho et al., 2012; Seillier et al., 2012).

## TP53INP1 Represses Tumor Cell Migration

One of the main features of cancer is the capability of cancer cells to migrate invasively through the stroma to form metastases, due to the significantly altered expression of the subset of genes involved in cell to cell and cell to extracellular matrix adhesion (Hanahan and Weinberg, 2011). TP53INP1 can work as a tumor suppressor by repressing tumor cell migration during metastasis. Secreted protein acidic and rich in cysteine (SPARC) regulates tumor cell-matrix interactions, and promotes cancer cell migration and metastasis (Podhajcer et al., 2008). SPARC gene expression is up-regulated in normal pancreas in the TP53INP1-deficient animals, and in pancreatic intraepithelial neoplasia lesions in a mouse model of pancreatic adenocarcinoma (Seux et al., 2011). TP53INP1 transcriptionally blocks SPARC gene expression, and silencing of TP53INP1 increases cell migration in mouse embryonic fibroblasts and pancreatic cancer cells (Seux et al., 2011). Consistent with these findings, miR-125b is overexpressed in type II endometrial carcinoma cells, and miR-125b expression increases endometrial carcinoma cell migration through down-regulating TP53INP1 expression (Jiang et al., 2011). Moreover, silencing TP53INP1 gene expression significantly correlates with lymphatic invasion in human gastric cancer patients (Jiang et al., 2006).

The role of TP53INP1 in suppressing cell migration is also of particular interest for cancer therapy, since the expression of genes involved in cell to cell and cell to extracellular matrix adhesion is significantly altered in some highly aggressive carcinomas (Hanahan and Weinberg, 2011), and TP53INP1 can potentially be an important player in invasion-metastasis cascade by targeting the genes. We propose that restoring TP53INP1 gene expression through targeting its silencers, such as miR-125, could effectively inhibit tumor invasion and metastasis.

## TP53INP1 Expression is Reduced in Human Tumor Tissues and Silencing of TP53INP1 Contributes to Tumorigenesis *In vivo*

Tumor protein 53-induced nuclear protein 1 protein expression has been found to be present in non-malignant human pancreatic lesions, but significantly or completely lost in the majority of primary pancreatic ductal adenocarcinomas and absent in metastatic tumors (Gironella et al., 2007). Consistently, significant reduction in TP53INP1 expression has also been detected in human gastric (Jiang et al., 2006) and colon cancer tissues (Shibuya et al., 2010). These observations demonstrate that reduction in TP53INP1 expression might be a general feature of tumor development. As knocking-out TP53INP1 gene expression promotes, and forced overexpression of TP53INP1 reduces, pancreatic and liver tumorigenesis in mice (Gironella et al., 2007; Ma et al., 2010), the restoration of TP53INP1 expression could be an effective approach for cancer therapy.

## TP53INP2 Exerts Effects Distinct from TP53INP1

Using a bioinformatics approach, Nowak et al. (2005, 2009) have identified TP53INP2, also known as diabetes and obesity regulated gene (DOR), as a TP53INP1-related gene. TP53INP2 encodes a protein with 30% amino acid identity and 45% similarity with TP53INP1, and shares two highly conserved regions (region 1: aa residues 28–42; region 2: 66–112 in human) with TP53INP1 (Sancho et al., 2012).

In spite of its homology with TP53INP1, TP53INP2 expression is not induced by p53, and forced overexpression of TP53INP2 does not alter the cell cycle or apoptosis. However, like TP53INP1, TP53INP2 is involved in the control of tumor development by modulating autophagy (Nowak et al., 2009). TP53INP2 functions as a scaffold protein, recruits LC3 and beclin-1 to the autophagosome through interacting with transmembrane protein vacuole membrane protein 1 (VMP1) (Nowak and Iovanna, 2009; Nowak et al., 2009), which is essential for autophagy (Ropolo et al., 2007). Upon autophagy-inducing stress such as starvation, TP53INP2 translocates from the nucleus to the autophagosomes, interacts with VMP1, recruits LC3, and beclin-1, but no beclin-2, and play an important role in autophagy (Nowak and Iovanna, 2009; Nowak et al., 2009) (Figure 2). Additionally, there are evidences suggesting that TP53INP1 and TP53INP2 can function as dual regulators of autophagy which make the proteins even more remarkable in controlling autophagy (Sancho et al., 2012).

TP53INP2 is also involved in tumor cell migration (Moran-Jones et al., 2009). Heterogenous nuclear ribonucleoprotein (hnRNPA2) is an important regulator of alternative splicing, is up-regulated in some invasive cancer types, and leads to tumor progression. It has recently been demonstrated that alternative splicing of exon 2 near the 5′ un-translated region of TP53INP2 is a key event downstream of hnRNPA2 that is necessary for cancer cells to migrate and invade through the extracellular matrix (Moran-Jones et al., 2009).

Despite the important roles of TP53INP2 in cancer cell autophagy and migration, it is not clear how TP53INP2 expression is controlled in cancer cells. Additionally, it is important to understand under which circumstances the splicing of TP53INP2 exon 2 by hnRNPA2 takes place, and whether hnRNPA2 also regulates alternative splicing of TP53INP1.

## Conclusion

Tumor suppressive functions of p53 and its homolog p73 reflect physiological activities of a wide range of their target genes. The identification and functional characterization of the critical gene/genes responsible for p53 and p73 induced tumor suppressive functions are very important for understanding tumorigenesis and for designing better cancer therapy.

Tumor protein 53-induced nuclear protein 1 gene expression is often silenced in tumor cells due to oncogenic factors such as the micro RNAs miR-93, miR-130b, miR-155, miR-125b, miR-17-5p, and miR-17, which down-regulate TP53INP1 expression through post-transcriptional mechanisms. Upon exposure to genotoxic agents, p53 and p73 activates TP53INP1 gene expression by directly binding to the TP53INP1 gene promoter. Additionally, the transcription factor E2F1 directly up-regulates TP53INP1 gene transcription independent of p53 and p73, and the inflammatory mediators tumor necrosis factor α and interleukin 6 also enhance TP53INP1 gene expression.

The TP53INP1 gene encodes two protein isoforms, TP53INP1α and TP53INP1β. Upon initial DNA damage, p53 is phosphorylated at Ser-15 and Ser-20, stimulating the binding of p53 to promoter regions of a subset of genes such as the G1 arrest genes p21, the DNA repair gene p53R2, and the p53 negative regulators such as MDM2. If DNA damage is severe, TP53INP1 forms protein complexes with the protein kinases HIPK2 or PKCδ to phosphorylate p53 at Ser-46, promoting the binding of p53 to the promoter regions of apoptosis related genes such as p53AIP1 rather than the repair related genes, leading to cell growth arrest and apoptosis (Figure 1). Additionally, TP53INP1 facilitates p73-mediated apoptosis independent of p53, enhances autophagic cell death by interacting with LC3, and represses tumor cell migration via regulating SPARC expression. As TP53INP1 expression is frequently silenced or completely lost in human cancer tissues, restoration of TP53INP1 expression could potentially inhibit tumor growth via its anti-proliferative, pro-apoptotic, pro-autophagic, and anti-cell migration activities. While TP53INP1 expression can be indirectly up-regulated by chemicals which activate p53 expression, we anticipate that small molecule compounds which activate TP53INP1 gene promoter activity through binding to p53-binding sites could potentially restore TP53INP1 expression, and the small molecule compounds could be discovered through screening small molecule compound libraries.

The TP53INP1-related gene TP53INP2 has recently been identified. Unlike TP53INP1, TP53INP2 expression is not modulated by p53, and TP53INP2 does not modulate cell cycle progression and apoptosis. While TP53INP2 induces autophagy via mechanisms similar to TP53INP1, an alternative splicing product of TP53INP2 RNA due to hnRNPA2 induces cancer cell migration. It is crucial to further understand how to control hnRNPA2-mediated TP53INP2 splicing to avoid cancer cell migration, and to retain TP53INP2-mediated autophagy, which synergizes with TP53INP1-induced autophagy. As such, restoration of TP63INP1 and TP53INP2 expression without the induction of hnRNPA2-mediated TP53INP1 RNA alternative splicing would ideally induce cancer cell cycle arrest, apoptosis, and autophagy, therefore simultaneously inducing binary cell death for a more effective therapy.

## Conflict of Interest Statement

The authors declare that the research was conducted in the absence of any commercial or financial relationships that could be construed as a potential conflict of interest.
